# Follicular lymphomagenesis: early steps and associated risk factors

**DOI:** 10.1186/1479-5876-10-S3-I10

**Published:** 2012-11-28

**Authors:** Sandrine Roulland

**Affiliations:** 1 Centre d’Immunologie de Marseille-Luminy, Aix-Marseille Université, Marseille, France; 2 INSERM U1104, Marseille, France; 3 CNRS UMR7280, Marseille, France

## 

Follicular lymphoma (FL) is a mature B-cell neoplasm resulting from the transformation of germinal center (GC) B-cells in secondary lymphoid organs. The acquisition of the t(14;18) chromosomal translocation (giving rise to a BCL2/IGH fusion and ectopic expression of the BCL2 proto-oncogene) constitute both a genetic hallmark and the critical early event in the natural history of FL [1]. However, as t(14;18) is detectable at low frequency (<1 per million B-cells) in up to 70% of healthy people, the relationship between t(14;18) and progression to disease remains unclear [2]. To date, the available data supports a multi-hit model of oncogenesis, where the stepwise acquisition of synergistic oncogenic events is required for full malignant transformation. The recent demonstration that memory B-cells can re-enter GCs and participate to new rounds of GC reactions has opened the possibility that multi-hit B-cell lymphomagenesis gradually occurs throughout life during successive immunological challenges[3-5]. Here we provide evidence for this scenario in FL using a sporadic BCL2^tracer^ mouse model mimicking FL’s hallmark t(14;18) translocation, combined with molecular/immunofluorescent tracking of t(14;18)^+^ clones and normal memory B-cells in paired lymphoid tissue samples from healthy individuals.

We show that BCL2-expressing memory B-cells require multiple GC transits to acquire the distinctive FL-like maturation arrest as GC B-cells with constitutive activation-induced cytidine deaminase activity, and to progress to advanced precursor stages.

This protracted process of GC co-opting, accumulating with age, would drive the major and early dissemination/progression of t(14;18)^+^ precursors observed in remote lymphoid tissues, including bone marrow, shaping the systemic disease presentation observed in most patients. Altogether, our data argue for a model of lymphomagenesis, in which progression to FL occurs asymptomatically over an extended period of time, by subverting the dynamic and plastic attributes of memory B-cells. Our characterization of the pre-clinical phases driving FL development in asymptomatic patients should help rationalize prospective approaches designed to identify biomarkers of risk, and innovative therapeutic targets present in early, potentially more curable phases of the disease.
